# PSMA-PET/CT-Positive Paget Disease in a Patient with Newly Diagnosed Prostate Cancer: Imaging and Bone Biopsy Findings

**DOI:** 10.1155/2017/1654231

**Published:** 2017-03-15

**Authors:** Michael Froehner, Marieta Toma, Klaus Zöphel, Vladimir Novotny, Michael Laniado, Manfred P. Wirth

**Affiliations:** ^1^Departments of Urology, University Hospital “Carl Gustav Carus”, Technische Universität Dresden, Fetscherstrasse 74, 01307 Dresden, Germany; ^2^Departments of Pathology, University Hospital “Carl Gustav Carus”, Technische Universität Dresden, Fetscherstrasse 74, 01307 Dresden, Germany; ^3^Departments of Nuclear Medicine, University Hospital “Carl Gustav Carus”, Technische Universität Dresden, Fetscherstrasse 74, 01307 Dresden, Germany; ^4^Departments of Radiologic Diagnostics, University Hospital “Carl Gustav Carus”, Technische Universität Dresden, Fetscherstrasse 74, 01307 Dresden, Germany

## Abstract

A 67-year-old man diagnosed with Gleason score 4 + 5 = 9 clinically localized prostate cancer with ^68^Ga-labeled prostate-specific membrane antigen-targeted ligand positron emission tomography/computed tomography (PSMA-PET/CT) positive Paget bone disease is described. Immunohistochemical staining revealed weak PSMA positivity of the bone lesion supporting the hypothesis that neovasculature might explain positive PSMA-PET/CT findings in Paget disease.

## 1. Introduction

^68^Ga-labeled prostate-specific membrane antigen-targeted ligand positron emission tomography/computed tomography (PSMA-PET/CT) is a valuable tool in the workup of patients with prostate cancer presenting with the suspicion of metastatic disease [1–3]. The sensitivity and specificity of PSMA-PET/CT for overall bone involvement in patients with prostate cancer have been found to be 99-100% and 88–100%, respectively [4]. In view of these high sensitivity and specificity values of this imaging modality, false-positive findings may create diagnostic pitfalls.

## 2. Case Presentation

**Figure 1:**
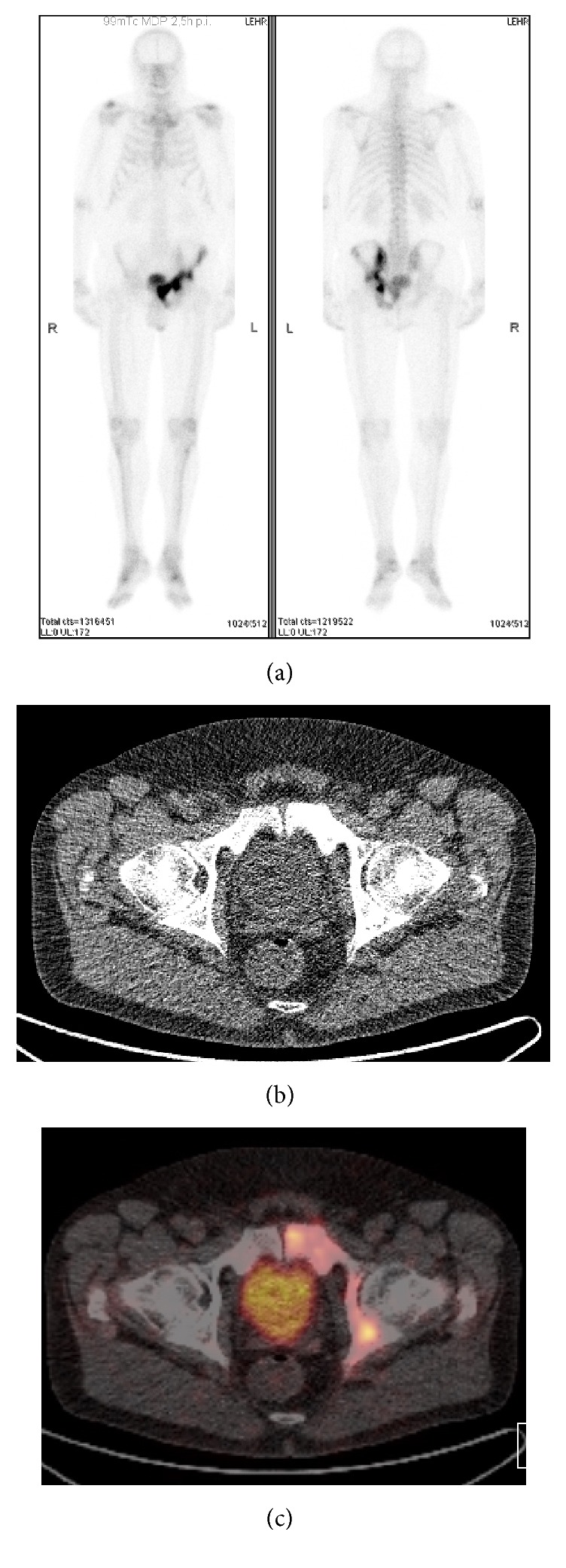
Bone scan showing increased uptake in the left-sided pelvis suggestive for Paget disease (a). Computed tomography demonstrated coarsened and bloated pubic bone (b). The lesion showed moderate uptake of ^68^Ga-labeled prostate-specific membrane antigen-targeted ligand (maximal standardized uptake value up to 13.8) (c). The maximal standardized uptake value of the primary tumor in the prostate was 10.0.

**Figure 2:**
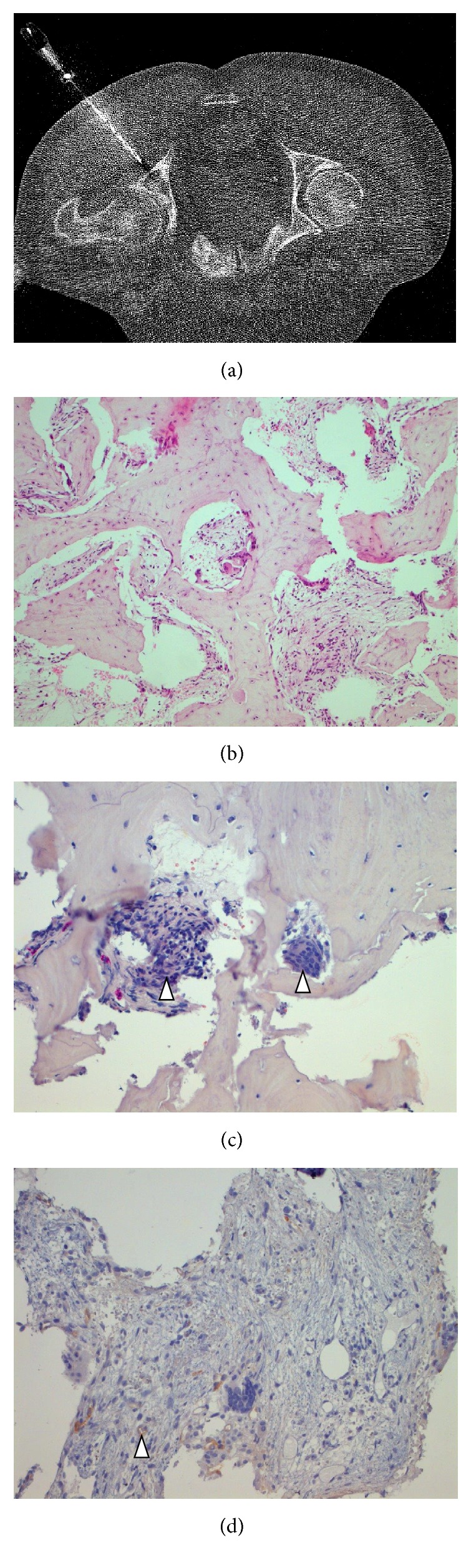
CT-guided biopsy (a) showed irregular bone structure with fibrotic marrow spaces ((b) H&E; original magnification ×20) containing multinucleated giant cells ((c) arrowheads; chloroacetate esterase stain; original magnification ×20). Immunohistochemical staining for PSMA revealed weak PSMA expression in endothelial cells ((d) arrowhead; original magnification ×20) in the Paget bone lesion.

An asymptomatic 67-year-old man was diagnosed with Gleason score  4 + 5 = 9  clinically localized prostate cancer (prostate-specific antigen, PSA, 6.7 ng/mL). A bone scan revealed increased pelvic tracer uptake that was considered suspicious for Paget disease (Figure 1(a)). ^68^Ga-labeled prostate-specific membrane antigen-targeted ligand positron emission tomography/computed tomography (PSMA-PET/CT) showed moderate PSMA positivity of this lesion (Figure 1(b)). Since Paget disease has been reported to cause PSMA positivity bone lesions [5–8], a bone biopsy was obtained for final workup that confirmed the diagnosis of Paget disease (Figure 2). After radical prostatectomy (pT3bpN0), PSA fell below the lowest detection level ruling out gross bone metastases. Six months after surgery, PSA was still undetectable and no symptoms of Paget disease were present.

## 3. Discussion

Paget disease is a common disorder of the skeleton characterized by hypertrophic and abnormally structured remodeling of bone [9, 10]. Many patients are asymptomatic, whereas others suffer from pain, nerve compression, or even pathologic factures. Rarely, malignant degeneration (osteosarcoma) may occur [9, 10]. Genetic and environmental factors play a role in the pathogenesis [10]. Bisphosphonates are used for treatment; it is, however, unknown whether they influence the national history of the disease [10].

Endothelial expression of PSMA in neovasculature known to occur in Paget disease has been postulated as the mechanism causing the PSMA-PET/CT positivity of this condition [5–8]. In the current case, we found some confirming evidence for this assumption with a weak PSMA positivity of endothelial cells in the bone affected by Paget disease (Figure 2(d)). Paget disease is a common disorder affecting up to 3% of senior adults [9]. PSMA-PET/CT positivity seems to be a usual phenomenon in Paget disease [5–8] that should be taken into consideration when PSMA-PET/CT is used during workup of patients with prostate cancer in order to avoid a pitfall in this otherwise accurate and sensitive diagnostic tool [1–3]. Beside Paget disease, various other tumors [11], coeliac ganglia [12], splenosis [13], sarcoidosis [14], and subacute stroke [15] have been reported to cause false-positive PSMA-PET imaging findings.
